# Effects of acute estradiol and progesterone on perimenstrual exacerbation of suicidal ideation and related symptoms: a crossover randomized controlled trial

**DOI:** 10.1038/s41398-022-02294-1

**Published:** 2022-12-30

**Authors:** Tory A. Eisenlohr-Moul, Savannah M. Bowers, Mitchell J. Prinstein, Katja M. Schmalenberger, Erin C. Walsh, Steven L. Young, David R. Rubinow, Susan S. Girdler

**Affiliations:** 1 Department of Psychiatry, The University of North Carolina at Chapel Hill, Chapel Hill, NC USA; 2 Department of Psychiatry, The University of Illinois Chicago, Chicago, IL USA; 3 Department of Psychology, The University of North Carolina at Chapel Hill, Chapel Hill, NC USA; 4 Institute for Medical Psychology, Heidelberg University Hospital, Heidelberg, Germany; 5 Department of Obstetrics and Gynecology, The University of North Carolina at Chapel Hill, Chapel Hill, NC USA

**Keywords:** Pathogenesis, Neuroscience

## Abstract

Female suicide attempts peak peri-menstrually—around the onset of menses—when the ovarian steroids estradiol (E2) and progesterone (P4) fall rapidly. Given preclinical evidence that withdrawal from either E2 or P4 can provoke behaviors consistent with elevated suicide risk, we hypothesized that withdrawal from one or both of these steroids contributes to perimenstrual exacerbation of suicidal ideation (SI) and related symptoms. In a randomized, controlled, double-blind crossover experiment (NCT03720847), a transdiagnostic sample of naturally cycling, medically healthy psychiatric outpatients reporting past-month SI completed two conditions during two different 14-day experimental intervals (days 7–20 where the luteinizing hormone surge = day 0), separated by a monthlong washout cycle. In the E2 and P4 (EP) condition, participants received transdermal E2 (0.1 mg/day) plus oral micronized P4 (200 mg/day as 100 mg twice daily) to buffer perimenstrual steroid withdrawal. A matched placebo (PBO) condition allowed natural perimenstrual steroid withdrawal. Participants reported daily SI and planning (primary outcomes) and indices of depression (low mood, hopelessness), threat sensitivity (anxiety, perceived stress), executive functioning (difficulty concentrating, impulsivity), and social cognitive bias (rejection sensitivity, perceived burdensomeness). In baseline cycles, no participant met prospective criteria for DSM-5 premenstrual dysphoric disorder, but 59% met all criteria except full follicular symptom remission, and 93% showed the highest SI in the perimenstrual phase. Of 29 randomized, 28 were analyzed (14 EP-PBO, 14 PBO-EP). Experimental administration of E2 and P4 (relative to PBO) reduced perimenstrual exacerbation of SI, suicide planning, depression, hopelessness, perceived stress, rejection sensitivity, and perceived burdensomeness, particularly in the perimenstrual (natural E2 and P4 withdrawal) days. Further, delayed withdrawal from experimental E2 and P4 (but not PBO) recapitulated SI, hopelessness, and rejection sensitivity. Acute perimenstrual withdrawal from ovarian steroids may play a causal role in perimenstrual worsening of depression and SI.

Suicide represents the fourth leading cause of death among females of reproductive age, accounting for 7% of deaths between 13 and 45 years [1]. Although more males die by suicide due to the selection of higher-lethality methods, females are twice as likely to experience suicidal thoughts or engage in suicidal behavior [2, 3]. The menstrual cycle, characterized by monthly fluctuations in estradiol (E2) and progesterone (P4), may play a role in this sex difference. At least 15 cross-sectional studies have reported a higher risk of suicidal ideation (SI), behavior, or death in the *perimenstrual* phase (broadly, within the 2 weeks surrounding menses onset) (for detailed reviews, see [4, 5]), with the greatest risk typically observed during menses. These weeks, characterized by dual elevation and subsequent rapid withdrawal from E2 and P4, may represent a time-varying biological trigger of acute suicide risk in many people. This heightened risk may result from detrimental impacts of cyclical ovarian steroid changes on diverse neurobehavioral pathways relevant to suicide, including depressed mood and hopelessness [6], poor executive functioning as reflected in difficulty concentrating or impulsive behavior [7–9], negative social appraisals such as rejection sensitivity or perceived burdensomeness to others [8, 9], or indices of threat sensitivity such as anxiety and perceived stress [10, 11].

Although most females do not experience notable affective, cognitive, or behavioral changes across the cycle, a large epidemiologic study demonstrated that 5.5% of the general female population met the criteria for DSM-5 premenstrual dysphoric disorder (PMDD) in daily ratings, showing at least five distressing or impairing neurobehavioral symptoms in the luteal phase that fully remit in the follicular phase ([12]; *N* = 1246 individuals). Another prospective epidemiologic study of those with depressive disorders found that 58% demonstrated significant premenstrual exacerbation (PME)—that is, *a degree of cyclical symptom change consistent with that required for PMDD that fails to meet DSM-5 criteria because symptoms do not remit fully in the follicular phase* (*N* = 58 individuals). Although small sample sizes preclude prevalence estimates for PME in most other psychiatric disorders, it is commonly observed (though not intrinsic) in many disorders, including borderline personality disorder [8, 9], bipolar disorder [13–15], eating disorders [16, 17], post-traumatic stress disorder [18], and panic disorder [19, 20]. In sum, many females with psychiatric disorders experience PME of their symptoms; this recurrent monthly worsening may reduce treatment response, increase severity and chronicity of symptoms over time, and increase the risk of suicidal thoughts and behaviors [21]. Currently, there is no DSM-5 diagnostic code or specifier for PME, and little research is available to guide treatment.

Despite the phenotypic overlap between PMDD and PME of depressive symptoms, a few studies point to possible differences in pathophysiology that should be further investigated. In PMDD patients, experiments implicate delayed effects of normal periovulatory steroid *surges* in luteal symptom provocation [22, 23], which can be mitigated by reversing or blocking steroid effects on GABAergic [24, 25] and serotonergic [26] function. However, these surge-sensitive steroid mechanisms do not uniformly trigger symptoms in PMDD patients despite similar symptom cyclicity in daily ratings (e.g., as in ref. [27]), and at least three studies have reported that those with PME of depressive disorders did not benefit from evidence-based PMDD treatments that work by producing low, stable endogenous steroid levels (oral contraceptives [28], GnRHa-induced chemical menopause [29]), or by antagonism of steroid metabolite activity at the GABA-A receptor [24]. These findings raise the possibility that PME of depression and related suicidality may be additionally or alternatively triggered by low levels of, or withdrawal from, E2 or P4.

Both preclinical and human studies demonstrate that ovarian steroid withdrawal (E2, P4, or both) can provoke affective, cognitive, and behavioral symptoms relevant to suicide in susceptible individuals. In euthymic patients with a history of menopausal-onset depression, blinded E2 withdrawal can recapitulate mood symptoms [30], and blinded, puerperal-like withdrawal from both E2 and P4 (in addition to perinatal-like surges) can similarly recapitulate symptoms in euthymic patients with a history of postpartum-onset depression [31, 32]. Further, many preclinical studies demonstrate that withdrawal from E2, P4, or both can induce low motivation and depression-like behavior [33, 34], emotion-related impulsivity [35], social avoidance or dysfunction [36], and threat sensitivity [37–39]. However, no human experiments to date have probed the effects of perimenstrual steroid withdrawal on menstrual cycle changes in SI, depressed mood, or related symptoms.

First, given the high prevalence of PME among patients with depressive disorders (~58%), we anticipated that most female patients with recent SI experience significant PME of affective symptoms, and would show peak SI in the days surrounding menses onset. Second, we hypothesized that perimenstrual withdrawal from E2, P4, or both plays a causal role in provoking PME of SI and related symptoms [4], and predicted that administration of both E2 and P4 in the weeks surrounding menses onset would reduce this cyclical symptom worsening. Combined administration of E2 and P4 was selected for this initial experiment to increase the external validity of the manipulation, since E2 and P4 naturally change together in the late luteal phase of the natural cycle. Given the chronicity of cyclical affective changes and the clinical complexity of patients with SI, we selected a crossover double-blind randomized controlled trial design in which patients could serve as their own controls.

## Patients and methods

The study is registered on clinicaltrials.gov (NCT03720847), including a full protocol with preregistered hypotheses as approved by the University of North Carolina at Chapel Hill Institutional Review Board prior to the enrollment of the first participant. All participants gave informed consent. Deidentified datasets and analytic code will be provided upon reasonable request to the corresponding author.

### Participants

To increase generalizability and reduce demand characteristics related to premenstrual syndrome and the menstrual cycle, participants were recruited from the community using social media advertisements “seeking participants for a study on the biology of depression, stress, and suicidal thoughts” (with no mention of sex, gender, hormones or the cycle). Eligibility criteria were assessed via online survey, phone, and at an enrollment visit: female sex, past-month SI but no past-month intent to act, and no attempts within the last year; seeing a licensed mental health professional at least once every 3 months; aged 18–45; no history of serious or chronic nonpsychiatric illness; no family or personal history of recurrent blood clots or known thrombophilia; predictable, regular menstrual cycles (25–35 days); not pregnant or breastfeeding and at least one year postpartum; not using hormonal medications or devices; BMI 18–39.99 kg/m^2^; not regularly smoking nicotine; no history of hospitalization for manic episode or psychosis, and no current substance use disorder (diagnosed at enrollment via SCID-5). Stable medication use was allowed and measured. Although participants were receiving outpatient mental health care, they were not receiving treatment from anyone affiliated with the study.

### Experimental design

**Fig. 1:**
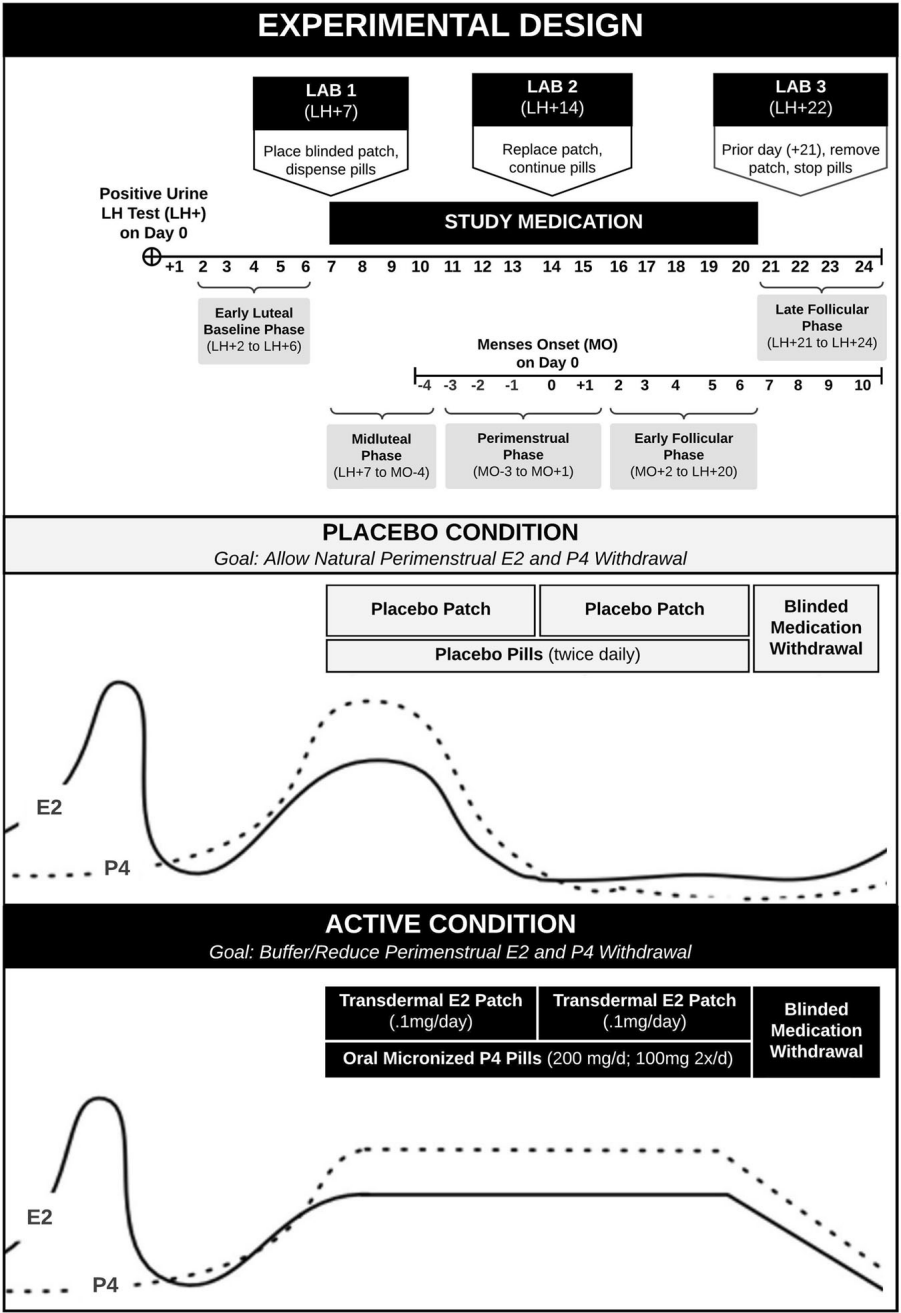
Crossover experimental design examining the effects of estradiol and progesterone administration in the perimenstrual (natural E2 and P4 withdrawal) phase of the cycle. In the “Experimental Design” panel, we outline (1) how at-home LH testing was used to schedule the experimental interval (study medication, laboratory visits), and (2) how cycle phases were coded during the experimental interval using both LH testing and self-reported menses onset. In the “Placebo Condition” and “Active Condition” panels, we describe the timing and other details of medication administration, along with hypothesized hormone levels in the two conditions.

To clarify the role of normal E2 and P4 withdrawal in the cyclical worsening of SI and related symptoms, we employed a randomized, placebo-controlled, double-blind (investigator, participant, assessor, but not data analyst), two-sequence crossover experiment (1:1 allocation ratio); Fig. 1 provides a visual overview of the experimental design and conditions. Here, “experimental interval” is used in lieu of the traditional label “study period” to avoid confusion with “menstrual period”. At an enrollment visit, participants were told, “the hormones given in this study are not being investigated as a treatment; instead, they are a tool to help us learn more about how the menstrual cycle might influence suicide risk in some people.” After the enrollment, participants provided a baseline menstrual cycle of daily symptom ratings, after which they completed two experimental intervals, bisected by a monthlong washout cycle, with each experimental interval timed to span 2 weeks surrounding expected menses onset (with medication taken from days +7 to +20 following a positive 40 mIU/ml urine luteinizing hormone test at-home, given that expected menses onset is approximately 13.3 days following the LH surge (day 0) [40]).

During each condition, participants attended lab visits on days LH +7, +14, and +22, where they provided a serum sample later assayed for E2 and P4 using radioimmunoassay (RIA). In the **placebo (PBO) condition**, participants received matched oral and transdermal placebos with the goal of observing symptom patterns under natural perimenstrual steroid withdrawal. In the **estradiol and progesterone (EP) condition**, participants received .1 mg/d transdermal E2 (Climara™; dosed as weekly patches at lab visits on day +7 and +14 following positive LH test) and 200 mg/d oral micronized P4 (Prometrium™; dosed 100 mg BID); the intention of this condition was to reduce the slope of natural perimenstrual E2 and P4 withdrawal (i.e., making it less steep), buffering the brain against the effects of abrupt perimenstrual withdrawal. The local investigational pharmacy managed randomization, blinding, and medication dispensation. Participants received twice-daily SMS pill reminders. The study was carried out at an off-campus clinical research facility affiliated with the university medical center. Participants were paid $600.

### Assessment schedule

At a 3-h enrollment visit, participants underwent the SCID-5 [41], SCID-PD [42], and C-SSRS [43] interviews. From enrollment to debriefing, participants reported on outcomes via daily online surveys (5 pm daily link delivered via SMS). During both experimental intervals (days +7 to +24 following positive LH test), daily phone calls (2 min per day) monitored adverse events, imminent suicide risk (including assessment of suicidal behavior), and medication compliance.

Original preregistered outcomes were based on less frequent lab-based interviews or self-reports; however, in the present paper, we chose to present the same outcomes *as reported on the daily self-report survey*, as recently recommended for alignment with best practice for the study of cyclical affective symptoms [44]. While the use of these more frequent daily assessments improves the granularity and interpretability of results, they do not result in substantive differences in the significance or direction of results (see NCT03720847 on ClinicalTrials.gov).

### Outcomes (measured via daily survey)

Eleven total outcomes from the daily survey were examined—two primary, five secondary, and four exploratory, as follows. **Primary outcomes** were daily SI and planning. Daily severity of suicidal ideation was measured by asking participants to rate their maximum past-24-h agreement (1 = Not at All to 5 = Extremely) with the statement, “I wanted to kill myself”, and severity of suicidal planning was measured as the mean of maximum past-24-h agreement with ASIQ items 3, 4, and 16 (“I thought about how I might kill myself”, “I thought about when I might kill myself”, and “I thought about ways people kill themselves”; Reliability: *R*_1F_ = 0.91 R_C_ = 0.69 [45]. **Secondary outcomes** as originally preregistered were depressed mood, hopelessness, impulsivity, anxiety, and feelings of social rejection. The daily record of severity of problems (DRSP) [46], the most commonly-used measure of hormone-sensitive affective symptoms, captured depressed mood (DRSP1: “felt depressed, sad, down, or blue”), hopelessness (DRSP2: “felt hopeless”), anxiety (DRSP4: “felt anxious, tense, ‘keyed up’, or ‘on edge’”), and rejection sensitivity (DRSP5: “was more sensitive to rejection or my feelings were easily hurt”). Impulsivity was measured using an adaptation of item 58 from the UPPS-P (“I did something impulsive that I might later regret“) [47]. **Exploratory outcomes** were perceived stress (Perceived Stress Scale item, “I felt unable to control the important things in my life”) [48], perceived burdensomeness (Interpersonal Needs Questionnaire item, “I felt that I was a burden to other people, or that they would be better off without me”) [49], difficulty concentrating (DRSP9: “had difficulty concentrating”), and anger/irritability (DRSP8: “felt angry or irritable”). Hormone-related physical symptoms were measured as a daily **covariate** (modified DRSP item: “I had physical symptoms, such as breast swelling or tenderness, joint or muscle pain, or bloating/weight gain”). Secondary, exploratory, and covariate items were rated on the DRSP response scale (1 = Not at All to 6 = Extreme). All non-DRSP items above were adapted from validated trait measures (as cited).

### Condition and cycle phase coding

See Fig. 1 for a visual depiction of experimental design, including laboratory visit scheduling, cycle phase coding, and a detailed explanation of experimental conditions. Days relative to positive luteinizing hormone test (LH+0) were coded such that day LH+1 corresponded to the day of ovulation, and so forth. Days relative to menses onset (MO + 0) were coded such that day the day before the onset of menses corresponded to day MO-1, and the second day of menstrual bleeding corresponded to MO+1. An experimental interval was defined as the 23 days starting the day after ovulation (LH+2, where LH+0 is the day of the positive urine LH surge test with a sensitivity of 40 mIU/ml) through the fourth day after stopping medication (LH+24, where the last day ON medication is LH 20). Each experimental interval day was coded for condition (0 = PBO or 1 = EP). Because we wanted experimental cycle phase (i.e., time) to have shared hormonal meaning between and within individuals despite natural variation in luteal phase lengths, we used both days relative to positive LH test and days relative to menses onset to code each experimental interval day as belonging to one of the following experimental cycle phases (see Fig. 1). First, the pre-medication early luteal phase was coded as days LH+2 through LH+6; in the normal cycle, these days generally correspond to rising E2 and P4. Second, the experimental midluteal phase was coded as the days from the start of medication (LH+7) through day MO-4; these days generally correspond to high/stable E2 and P4. Third, the experimental perimenstrual phase was coded as days −3, −2, −1, 0, and 1 relative to menses onset (day 1); these days generally correspond to the steep E2 and P4 withdrawal that triggers the onset of menses [40, 50]. Fourth, the experimental early follicular phase was coded as the days from day 2 following menses onset (MO+2) through the final day of medication (LH+20); these days generally represent low E2 and P4. Fifth, and finally, the medication withdrawal days of the post-medication late follicular phase (phase = 5) were coded as the first day off study medication through the end of the experimental interval (LH+21 to LH+24); in the normal cycle, these days generally represent higher and rapidly rising E2. Thus, each phase was coded to best preserve its hormonal meaning; most importantly, the five-day perimenstrual phase, which occurred fully within the 2 weeks of medication administration in all cases, corresponded to endogenous steroid withdrawal across all experimental intervals.

### Statistical analyses and power

Initial descriptive analyses utilized the C-PASS [51], a standardized method for diagnosing PMDD, to determine the percentage of the sample showing a PMDD symptom pattern in the baseline cycle of daily symptom ratings. Manipulation checks and hypothesis tests were carried out in three-level multilevel models (daily observations at level 1, nested within experimental interval at level 2, nested within participants at level 3). We expected to observe a symptom increase from the early luteal to the perimenstrual phase under PBO that would be prevented in the EP condition. In statistical terms, we predicted an interaction of experimental condition (at level 2) with cycle phase contrast (between early luteal and perimenstrual; at level 1) predicting daily outcomes. Daily physical symptoms were centered around the individual’s mean and covaried. Analyses proceeded in SAS PROC MIXED utilizing maximum likelihood estimation and Kenward-Roger degrees of freedom. Random intercepts were included at the person and condition levels. A priori power calculations based on the expectation of conventionally medium (*f* =0.25) within-person experimental effects and significant outcome clustering (ICC ~0.60) in a repeated measures context indicated that 28 completers would be required to achieve 80% power. We sought to achieve 30 completer participants. The Holm–Bonferroni method was used to adjust for multiple tests.

## Results

### Participant flow

**Fig. 2:**
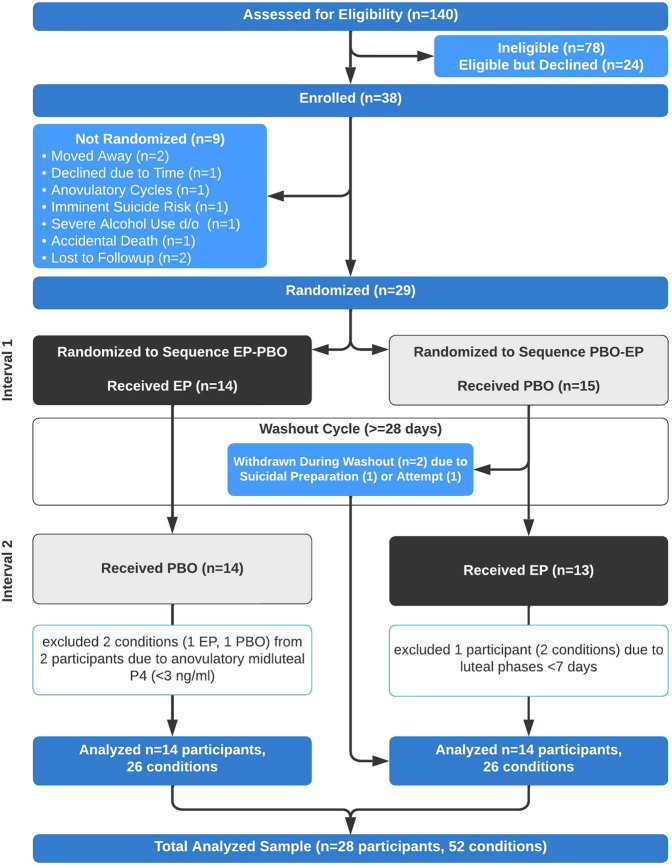
CONSORT diagram for the crossover two-interval randomized clinical trial.

Recruitment began in December 2016 and ended in July 2017; data collection began 1 January 2017 and concluded 15 October 2017. Figure 2 (CONSORT) illustrates participant flow. Of 140 screened, 38 were enrolled, 29 were randomized. Of 15 randomized to the PBO-EP sequence, 14 were analyzed (26 phases); two were withdrawn during washout due to self-reported suicide attempt (*n* = 1) and preparation for suicide attempt (*n* = 1) (both following PBO, both occurring within 7 days of menses onset); and one additional participant was excluded from analysis because their menses started prior to the start of medication (i.e., positive LH+7) in both experimental intervals. All 14 randomized to the EP-PBO sequence completed both experimental conditions and were included in analyses (26 phases); two phases (one each from two people) were excluded due to midluteal P4 (< 3 ng/ml) consistent with anovulation. The final analyses included 28 participants (52 experimental intervals; 1122 daily surveys).

### Baseline characteristics

Table 1 reports demographic, diagnostic, and treatment characteristics in the final sample and by sequence. Those randomized to the EP-PBO sequence were younger, less likely to be cohabitating with a partner, and more likely to meet the criteria for current post-traumatic stress disorder. Covarying these factors did not substantively alter hypothesis tests. Regarding stable concomitant treatments, 12 participants (42.9%) were taking stable selective serotonin reuptake inhibitors (SSRIs), 3 were taking stimulant medications, 3 were taking mood stabilizing medications, and 50% reported weekly psychotherapy visits. Upon the recommendation of a reviewer, SSRI use was considered as a covariate, but was removed given that it was not a significant predictor of any symptom, and its inclusion did not alter the direction or significance of any hypothesized effect.

**Table 1:** Participant characteristics in the full sample and by sequence.

	Total sample (*N* = 28)		PBO-EP (*N* = 14)		EP-PBO (*N* = 14)	
	*n*	%	*n*	%	*n*	%
Age, years, mean (SD)^a^	28.7 (6.8)		32.1 (6.6)		25.5 (5.6)	
BMI at recruitment (SD)	26.3 (6.1)		28.2 (6.3)		24.5 (5.3)	
White race	25	89.2	12	85.7	13	92.9
Education						
<4 yr college degree	4	14.2	1	7.1	3	21.4
4 yr college degree	15	53.6	8	57.1	7	50.0
Post-graduate degree	9	32.1	5	35.7	4	28.5
Household income						
Less than $25,000	8	28.6	1	7.1	7	50.0
$25,000–$49,999	8	28.6	3	21.4	5	35.7
$50,000–$99,999	8	28.6	8	57.1	0	0.0
$100,000 or more	4	14.3	2	14.2	2	14.2
Sexual orientation						
Heterosexual	11	39.2	6	42.8	5	35.7
Bisexual	8	28.6	3	21.4	5	35.7
Lesbian/gay	3	10.7	0	0.0	3	21.4
Other non-heterosexual	6	21.4	5	35.7	1	7.1
Gender (Cisgender)	26	92.8	12	85.7	14	100
Cohabitation with partner^a^	18	64.3	12	85.7	6	42.8
Parity	9	32.1	5	35.7	4	28.7
Current daily SSRI use	12	42.9	7	50.0	5	35.7
Current daily stimulant use	3	10.7	2	14.2	1	7.1
Current daily mood stabilizer use	3	10.7	1	7.1	2	14.2
Current weekly psychotherapy	14	50.0	6	42.8	8	57.1
Lifetime non-suicidal self-injury	17	60.7	7	50.0	10	71.4
Lifetime suicide attempt	16	57.1	8	57.1	8	57.1
Any depressive disorder	22	78.6	12	85.7	10	71.4
Major depressive disorder	19	67.9	11	78.6	8	57.1
Persistent depressive disorder	19	67.9	10	71.4	9	64.3
Attention deficit/hyp disorder	4	14.3	2	14.3	2	14.3
Generalized anxiety disorder	13	46.4	7	50.0	6	42.9
Social anxiety disorder	14	50.0	7	50.0	7	50.0
Panic disorder	2	7.1	1	7.1	1	7.1
Agoraphobia	3	10.7	3	21.4	0	0.0
Specific phobia	7	25.0	4	28.6	3	21.4
Obsessive–compulsive disorder	4	14.3	3	21.4	1	7.1
Anorexia nervosa	1	3.6	0	0.0	1	7.1
Bulimia nervosa	1	3.6	1	7.1	0	0.0
Post-traumatic stress disorder^b^	9	32.1	2	7.1	7	50.0
Borderline PD	7	25.0	5	35.7	2	14.2
Avoidant PD	6	21.4	4	28.6	2	14.3
Obsessive–compulsive PD	2	7.1	0	0.0	2	14.3
Lifetime physical/sexual abuse	15	53.6	8	57.1	7	50.0
Abuse before age 13	5	17.9	2	14.3	3	21.4
C-PASS MRMD (BL cycle)	0/27	0.0	0/14	0.0	0/13	0.0
C-PASS PME (BL cycle)	16/27	52.9	8/14	57.1	8/13	61.5

^a^PBO-EP > EP-PBO; ^b^EP-PBO > PBO-EP.

*BL* baseline, *PD* P-personality disorder, *BMI* body mass index, *MRMD* menstrually related mood disorder, as defined by the C-PASS.

### Baseline premenstrual disorder diagnosis

C-PASS-compatible baseline data were available for 27 of the 28 participants. None of these participants met C-PASS criteria a cycle-level diagnosis of PMDD (nor C-PASS “MRMD”). However, when removing the requirement of *absolute clearance* (i.e., that all postmenstrual symptoms must be less than moderate, <4), utilized previously to diagnose PME [9], 16 participants (59.2%) met criteria on at least one core emotional symptom (DRSP1–8). This is consistent with prior prospective estimates of 58% with PME among females with a depressive disorder [6]. 25 participants (92.6%) demonstrated their own maximum levels of SI in the perimenstrual phase.

### Adherence and missingness

Study adherence was high; among those analyzed, 86.8% of daily surveys were completed, and there were no missing visits. Daily medication compliance was monitored via daily phone call; on average, participants reported missing less than one pill per condition, and all patches that fell off prematurely were replaced within 24 h. A logistic multilevel model predicting survey missingness from condition indicated a trend toward lower likelihood of survey missingness (i.e., greater compliance) in the EP condition (*Est* = −0.38, SE = 0.21, *t*(1167) = −1.80, *p* = 0.07; OR = 0.68).

### Hormone levels

The EP condition buffered the slope of midluteal-to-perimenstrual steroid withdrawal; there was a significantly slower midluteal-to-perimenstrual decline in both E2 (*Condition* × *Phase Est* = 51.15, SE = 18.56, *t*(91.2) = 2.75, *p* = 0.007) and P4 (*Est* = 14.19, *SE* = 5.70, *t*(93.9) = 2.49, *p* = 0.014) in the EP condition (Supplemental Fig. 1).

### Luteal phase length, menstrual bleeding, and physical symptoms

Luteal phase length (days between LH surge and menses onset) did not significantly differ by condition (EP-PBO, Mean Difference = 0.88, *SE* = 0.55, *t*(23) = 1.57, *p* = 0.13). However, there was an extension of menstrual bleeding in the EP condition, apparently due to withdrawal from experimentally administered E2 and P4 (days LH 21–24; Supplemental Fig. 2). Participants showed a greater persistence of physical symptoms from early luteal to the early and late follicular phases in the EP condition, likely due to the extension of menses (see Table 2).

**Table 2:** Three-level regression models testing effects of EP on cyclical exacerbation of suicidality and related symptoms.

	Suicidal ideation severity		Suicidal planning severity		Depressed mood (DRSP)		Hopelessness (DRSP)		Impulsivity (UPPS)		Difficulty concentrating (DRSP)	
	Est.	SE	Est.	SE	Est.	SE	Est.	SE	Est.	SE	Est.	SE
Intercept	**1.213**	0.071	**1.147**	0.038	**2.338**	0.161	**2.203**	0.193	**1.503**	0.105	**2.278**	0.163
Daily physical symptoms (person-centered)	0.036	0.021	**0.047**	0.011	**0.150**	0.039	**0.078**	0.028	**0.056**	0.028	**0.206**	0.036
Condition (Ref=PBO)	0.139	0.081	0.001	0.041	0.087	0.153	−0.025	0.150	−0.114	0.107	0.047	0.129
Midluteal (vs EL)	0.023	0.046	−0.032	0.024	**0.342**	0.084	0.093	0.085	−0.052	0.059	−0.004	0.079
Perimenstrual (vs EL)	**0.167**	0.045	0.040	0.023	**0.272**	0.082	**0.175**	0.083	−0.040	0.058	0.026	0.077
Early Follicular (vs EL)	**0.149**	0.047	**0.052**	0.024	**0.184**	0.086	0.110	0.089	0.014	0.061	**0.200**	0.081
Late Follicular (vs EL)	0.071	0.049	0.026	0.025	**0.184**	0.089	0.055	0.091	0.084	0.062	**0.268**	0.083
Condition × midluteal (vs EL)	−0.118	0.064	0.009	0.033	**−0.322**	0.117	−0.173	0.116	0.001	0.082	−0.070	0.109
Condition × perimenstrual (vs EL)	−0.275*	0.064	−0.060*	0.022	−0.233*	0.116	−0.243*	0.097	−0.063	0.082	−0.092	0.109
Condition × early follicular (vs EL)	**−0.144**	0.069	**−0.085**	0.035	−0.200	0.125	−0.115	0.126	0.063	0.088	**−0.348**	0.117
Condition × late follicular (vs EL) *[Medication Withdrawal Phase*]	**0.205**	0.070	0.026	0.035	0.035	0.123	**0.186**	0.083	0.061	0.087	−0.097	0.116
Covariance parameters												
Intercept (person-level)	**0.050**	0.024	**0.019**	0.009	**0.406**	0.173	**0.724**	0.234	**0.151**	0.072	**0.511**	0.171
Intercept (condition-level)	**0.054**	0.018	**0.013**	0.005	**0.195**	0.073	**0.189**	0.062	**0.094**	0.036	**0.124**	0.044
Residual (day-level)	**0.125**	0.005	**0.032**	0.001	**0.413**	0.018	**0.374**	0.017	**0.205**	0.009	**0.362**	0.015

	Rejection sensitivity (DRSP)		Perceived burdensomeness (INQ)		Anxiety (DRSP)		Perceived stress (PSS)		Anger/irritability (DRSP)		Physical symptoms (DRSP)	
	Est.	SE	Est.	SE	Est.	SE	Est.	SE	Est.	SE	Est.	SE
Intercept	**1.876**	0.145	**1.835**	0.189	**2.580**	0.138	**1.992**	0.165	**2.018**	0.120	**1.427**	0.095
Daily physical symptoms (person-centered)	**0.128**	0.035	**0.137**	0.029	0.076	0.039	**0.097**	0.035	**0.077**	0.039		
Condition (Ref=PBO)	−0.101	0.142	0.047	0.104	−0.158	0.132	0.126	0.075	−0.191	0.125	0.000	0.134
Midluteal (vs EL)	0.070	0.076	−0.029	0.064	0.033	0.084	**0.262**	0.073	0.133	0.084	−0.039	0.065
Perimenstrual (vs EL)	**0.102**	0.024	0.034	0.062	−0.058	0.082	**0.318**	0.077	0.050	0.082	**0.164**	0.063
Early follicular (vs EL)	0.069	0.078	**0.139**	0.065	−0.023	0.086	0.152	0.079	−0.129	0.086	**−0.144**	0.066
Late follicular (vs EL)	−0.117	0.080	−0.004	0.067	−0.070	0.089	0.055	0.091	−0.101	0.089	**−0.257**	0.068
Condition × midluteal (vs EL)	−0.097	0.105	−0.100	0.088	0.151	0.117	**−0.284**	0.104	**−0.241**	0.117	0.056	0.088
Condition × perimenstrual (vs EL)	−0.193*	0.0805	−0.227*	0.088	0.283	0.206	−0.319*	0.103	0.056	0.116	0.104	0.089
Condition × early follicular (vs EL)	−0.067	0.113	**−0.276**	0.095	**0.378**	0.125	**−0.453**	0.112	**0.394**	0.125	**0.459**	0.095
Condition × late follicular (vs EL) *[Medication Withdrawal Phase*]	**0.252**	0.111	0.050	0.093	0.294	0.224	0.150	0.110	**0.371**	0.124	**0.244**	0.094
Covariance parameters												
Intercept (person-level)	**0.313**	0.134	**0.854**	0.254	**0.296**	0.109	**0.491**	0.172	**0.188**	0.079	**0.098**	0.038
Intercept (condition-level)	**0.172**	0.062	**0.079**	0.028	**0.122**	0.043	**0.171**	0.058	**0.103**	0.039	**0.046**	0.018
Residual (day-level)	**0.336**	0.014	**0.236**	0.010	**0.415**	0.018	**0.328**	0.014	**0.415**	0.018	**0.252**	0.011

Bolded values indicate significance at *p* < 0.05. Asterisks indicate primary hypothesis contrasts that are statistically significant after correction for multiple outcomes using the Holm–Bonferroni Method.

### Participant blinding

After 7 days on medication in each condition (LH+14), participants rated their agreement with the statement, “In the past week, I thought I was taking active study medication” on a visual analog scale (0–100). Ratings did not differ significantly by condition within a given participant, indicating successful blinding (EP-PBO, Mean Paired Difference = 5.76, *SD* = 43.18, *t*(23) = 0.611, *p* = 0.55).

### Hypothesis tests

**Fig. 3:**
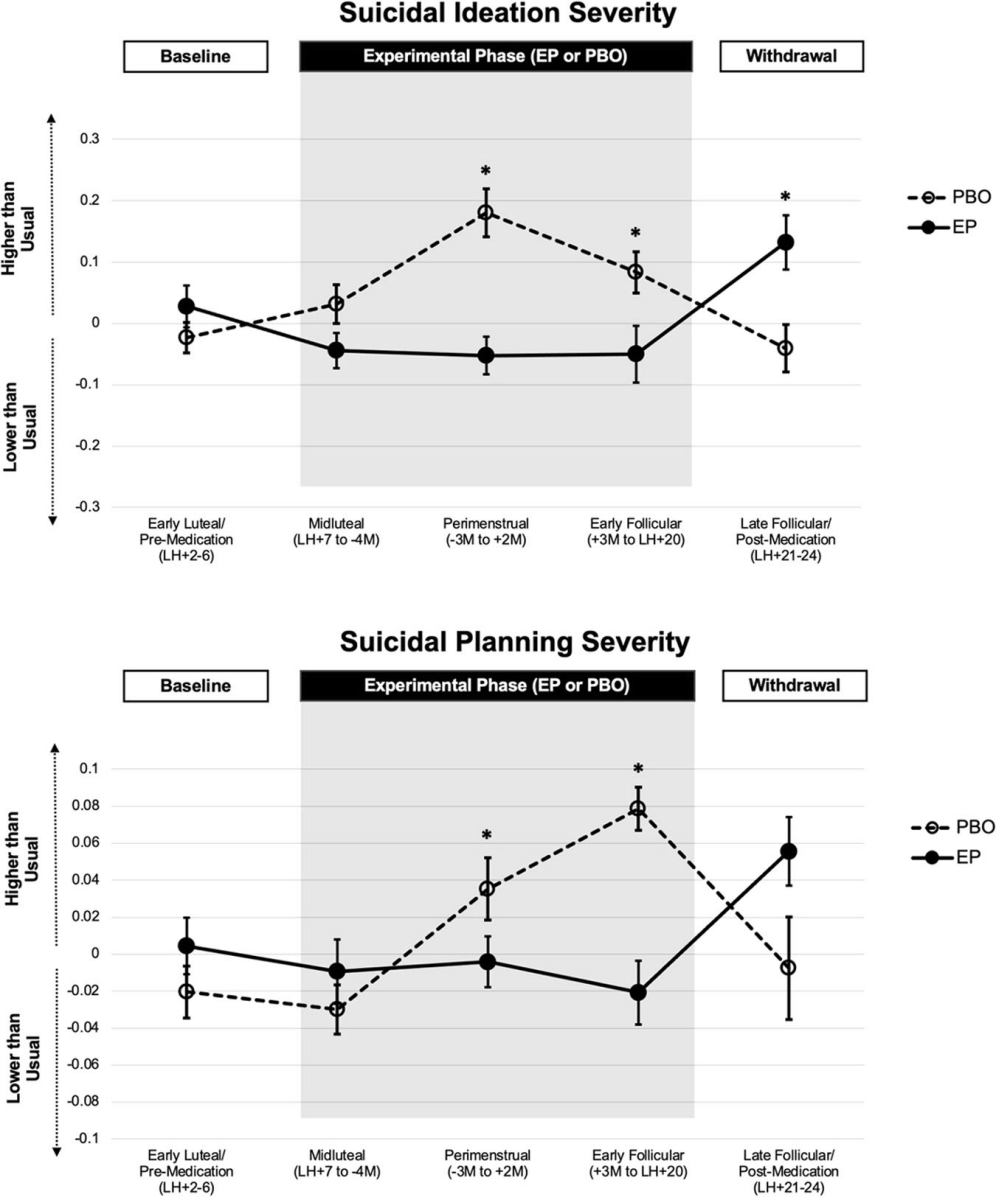
Graphical depiction of primary suicidal ideation and planning results. Asterisks indicate significant condition × phase interaction effects. Error bars represent SEM.

Table 2 presents hypothesis test models. Figure 3 illustrates primary outcomes, and Supplemental Fig. 3 illustrates additional outcomes. Our prediction was a statistical interaction of the Condition and Experimental Cycle Phase predicting all symptoms, such that symptoms would increase from the early luteal to the perimenstrual phase under PBO (during acute perimenstrual steroid withdrawal), but this PME would be prevented in the EP condition. CONSORT guidelines advise against statistical tests for crossover effects [52]; however, visual inspection revealed a similar pattern of experimental effects in both the first and second experimental intervals.

#### Primary suicide-related outcomes

Consistent with the hypothesis that acute perimenstrual withdrawal from ovarian steroids contributes to PME of SI, significant Condition × Phase interactions were observed for both suicide outcomes. *SI and planning increased from the early luteal to the perimenstrual phase in the PBO condition, but the EP condition prevented this increase*. For both outcomes, this experimental benefit of E2 and P4 administration was absent in the earlier experimental midluteal phase but present in both the perimenstrual and early follicular phases. For SI only, there was also a significant interaction representing recapitulation of risk in the late follicular phase of the EP condition, when exogenous steroids were withdrawn.

#### Secondary and exploratory outcomes

Significant Condition × Phase interactions consistent with the predicted perimenstrual benefit of the EP condition relative to PBO were also observed for depressed mood, hopelessness, perceived stress, rejection sensitivity, and perceived burdensomeness, but not for anxiety, difficulty concentrating, impulsivity, or anger/irritability. Benefits of E2 and P4 administration persisted into the early follicular phase for perceived stress and perceived burdensomeness, and an additional benefit was observed in this phase for difficulty concentrating. As with SI, hopelessness and rejection sensitivity showed a recapitulation of risk during exogenous steroid withdrawal in the late follicular phase of the EP condition.

Several unexpected Condition × Phase effects were also present. Both depressed mood and perceived stress showed a benefit of the EP condition in the earlier midluteal phase, when endogenous steroids have not yet abruptly withdrawn, suggesting that the benefit to these outcomes may not be specific to buffering of E2 and P4 withdrawal. Additionally, the EP condition was associated with a worsening of anxiety and anger/irritability in the early follicular phase, and this worsening of anger/irritability persisted into the late follicular phase.

### Adverse events

Overall, adverse events were mild to moderate and similar across conditions; the most common included breast tenderness and patch-related skin itching or irritation. There were no life-threatening adverse events during experimental intervals. Two participants reported distressing changes in irritability and anxiety in the EP condition that were (1) associated with functional impairment, (2) not present in the PBO condition, and (2) temporally related to study medication. When these two participants were excluded from hypothesis tests in a sensitivity analysis, all significant Condition × Phase effects for Anger/Irritability and Anxiety outcomes became nonsignificant, suggesting that the adverse responses observed in the late experimental phases for those outcomes were driven primarily by these participants’ responses.

## Discussion

E2 and P4 administration timed to the two weeks surrounding menses onset prevented premenstrual worsening of SI and planning, as well as depressed mood, hopelessness, rejection sensitivity, perceived burdensomeness, and perceived stress, with some evidence of an additional benefit for difficulty concentrating in the subsequent early follicular phase. The benefits of E2 and P4 administration were most marked in the perimenstrual phase (days −3 to +2 surrounding menses onset), when the majority of E2 and P4 withdrawal occurs, and were most often also present in the early follicular phase, just following the acute endogenous steroid withdrawal that initiates menstrual bleeding. However, these effects were also present for depressed mood and perceived stress in the midluteal phase, prior to the initiation of endogenous steroid withdrawal, which suggests the possibility of an additional or alternative mechanism of benefit (in addition to buffering the effects of E2 and P4 withdrawal). For SI, hopelessness, and rejection sensitivity, there were recapitulations of the E2 and P4 withdrawal effect during withdrawal from exogenous hormones in the active condition, underscoring a causal role for withdrawal. Consistent with prior epidemiologic studies, 59% of the sample demonstrated PME of at least one emotional symptom in the baseline phase, with nearly all showing their highest baseline levels of SI in the perimenstrual phase. Two participants were withdrawn in the washout phase due to suicide attempt (*n* *=* 1) or preparation (*n* = 1), and in both cases these behaviors occurred in the 7 days surrounding onset of menstrual bleeding. Together, these results support a causal, contributory role of cyclical ovarian steroids—and especially E2 and P4 withdrawal—in premenstrual exacerbation of SI and related symptoms among cycling individuals with suicidality. These results elucidate one of the first modifiable, time-varying biological triggers for suicidality.

### Possible mechanisms of steroid-withdrawal-related suicidality

#### Behavioral mechanisms

In the present study, we observed benefits of dual E2 and P4 administration (vs natural hormone changes under placebo) on depressed mood, hopelessness, perceived stress, rejection sensitivity, perceived burdensomeness, and difficulty concentrating. Since depressed mood, hopelessness, perceived social alienation/burdensomeness, and situational entrapment/stress are each robustly associated with suicidality [53], future work should investigate these pathways more closely to determine their mechanistic underpinnings and develop related treatments. Of note, experiences of overwhelm and stress (i.e., Research Diagnostic Criteria (RDoC) sustained threat), but not anxiety (RDoC potential threat), were improved by perimenstrual administration of E2 and P4. This finding should be replicated and extended using tasks and psychophysiological methods to confirm this divergent pattern and examine its relevance for understanding the pathophysiology of steroid-withdrawal-induced symptoms [54]. Contrary to our hypotheses, we did not observe robust experimental effects on self-reported indices of executive functioning, except for an early follicular benefit on difficulty concentrating. It is possible that only some individuals experience cyclical worsening of cognitive function, and more work is needed using objective markers to evaluate this pathway.

It is striking that three of the significant effects—hopelessness, increased sensitivity to social rejection, and perceived burdensomeness to others—were significant only during periods of endogenous or exogenous steroid withdrawal, which may indicate a unique effect of withdrawal on cognitive biases that increase risk for both affective symptoms and suicide. Increased hopelessness represents a depressive cognitive bias in which suffering is interpreted as permanent. Increased perceptions of burdensomeness to other people are usually a cognitive bias in which social support is perceived as limited, or as coming at the expense of others’ welfare. Rejection sensitivity is another depressive cognitive bias in which one perceives social rejection in the absence of actual rejection or has more intense feelings of social rejection than are warranted by the social context. Although it is theoretically possible that these cyclical changes in cognitive appraisal represent accurate perceptions that the environment is somehow changing with the menstrual cycle, it seems far more likely that these changes reflect the effects of perimenstrual steroid withdrawal on emotion-related cognitive processes, which are broadly implicated in affective disorders—especially those characterized by suicidality [55].

#### Biological mechanisms

The present study was not designed to disentangle the unique effects of E2 and P4 withdrawal, nor to explicate the molecular or circuit-level mechanisms by which steroid withdrawal may influence symptoms. Nevertheless, several candidate pathways deserve mention.

First, perimenstrual withdrawal from the normative beneficial effects of GABAergic neuroactive steroid metabolites of P4 (e.g., 3α,5α-THP; “allopregnanolone”) may be involved. For many, cyclical symptom changes may result from an exaggerated response to withdrawal from the antidepressant or anxiolytic effects of 3α,5α-THP, and other GABAergic P4 metabolites. This rapid withdrawal from 3α,5α-THP in the several days surrounding menstrual onset is driven primarily by withdrawal from their precursor (P4), but may also be influenced by withdrawal from E2, which alters the expression of enzymes related to the formation of neuroactive steroids [56, 57]. Both E2 and P4 withdrawal could be expected to lead to abrupt steroid changes in brain circuits implicated in emotion processing and regulation [58]. Second, preclinical studies indicate that steroid surges and withdrawal are capable of altering the structure and function of GABAAR, leading to a reversal of typical inhibitory effects [39], which are frequently hypothesized to contribute to PMDD. Consistent with this notion, the use of full-cycle dutasteride to stabilize fluctuations in 5α-reduced steroid metabolites may mitigate PMDD symptoms even when precursor fluctuations persist [25]. Third, perimenstrual withdrawal from the myriad effects of E2 on neurotransmitters and other signaling molecules may trigger affective and cognitive dysregulation. E2 regulates the synthesis and activity of serotonin, dopamine, and norepinephrine, along with a host of other molecules, including neurotrophic and immune factors (see [58] for a comprehensive review). Any one of these mechanisms, or their combinations, may be relevant. Both E2 and P4 withdrawal can alter the functioning of brain circuits implicated in emotional experience as well as appraisal of situations, stressors, and social information, and these changes may underlie observed effects.

### Comparisons to the pathophysiology of other reproductive mood disorders

Our findings are consistent with experimental work demonstrating that steroid withdrawal can trigger affective symptoms in several other lifespan reproductive mood disorders, including perimenopausal-onset depression [30] and postpartum-onset depression [31, 32, 59]. However, the E2 and P4 withdrawal mechanism tested in the present study can be contrasted with those observed in prototypical PMDD (i.e., luteal phase confinement of significant affective symptoms), which is robustly triggered not by proximal hormone withdrawal [23] but rather by the delayed effects of periovulatory steroid surges [22]. It should be noted that, despite the fact that suicidality was not included in the diagnostic criteria for DSM-5 PMDD, and despite luteal phase confinement of symptoms in PMDD, recent studies have demonstrated a high prevalence of SI (40% current [60], 71% lifetime) and suicide attempts (34% lifetime [61]) among patients with prospectively-diagnosed PMDD. Given that perimenstrual suicidality is also commonly present in PMDD (i.e., E2 and P4 surge-related perimenstrual symptoms), E2 and P4 withdrawal probably represents just one of several steroid change triggers that are capable of increasing suicidality among hormone-sensitive individuals.

More experimental and epidemiologic work is needed to delineate the nature of specific types or dimensions of hormone sensitivity in reproductive psychiatric disorders [62, 63]. This may eventually lead to a more basic, mechanistic understanding of hormone-sensitive symptoms that make distinctions between PMDD and PME unnecessary. Building such a transdiagnostic, dimensional model of hormone sensitivity could foster the development of more precise diagnoses and treatments [63, 64].

#### Strengths, limitations, and future directions

This trial had many strengths. First, the experimental crossover design provides the clearest evidence to date implicating E2 and P4 withdrawal in the increased suicide risk accompanying perimenstrual affective changes. Second, the use of sophisticated multilevel models improved precision of our results, as it allowed us to explore the specificity of E2 and P4 withdrawal effects. Third, the transdiagnostic outpatient sample, not recruited for perceived premenstrual symptoms, generalizes readily to outpatient samples. Fourth, the use of at-home urine LH testing and serum P4 to confirm ovulation and timing of the hormone administration increased the precision of our experiment.

The trial also had significant limitations. First, the study was powered to detect conventionally medium-sized effects, and therefore may have failed to detect smaller effects. Second, the sample was not selected for PME of SI. While this was done to reduce demand characteristics and allow for dimensional study of the phenomena in question, it led to more modest effect sizes in the full sample than could be observed in the raw data of some patients with more severe PME. Future work focused on treatment development must recruit participants for the presence of cyclical symptom change in daily ratings. A larger sample is needed to determine whether the benefits described here are consistent across participants, or whether unique risk factors identifiable at baseline may predict response. Third, although we used hormonal preparations identical to endogenous E2 and P4, we cannot rule out the possibility that E2 and P4 administration was beneficial due to elevated levels of metabolites. Oral micronized P4 is extensively metabolized to 3α,5α-THP and other GABAergic neurosteroids during first-pass metabolism [65]. Fourth, the present design does not allow us to differentiate the effects of E2 vs P4 withdrawal on perimenstrual symptom change; it is possible that the benefits observed in this study are attributable primarily or solely to one of the two steroids. Future studies should compare administration of E2 and P4 to determine whether either individually produce a beneficial effect, or whether administration of both hormones is required to reduce PME of SI and related symptoms. Finally, this study could not examine suicide attempts as an outcome given the low base rate of this behavior, which may have been due in part to the daily study phone calls monitoring imminent suicide risk.

## Conclusions

Cyclical hormone flux—and perimenstrual E2 and P4 withdrawal specifically—appears to contribute to the perimenstrual exacerbation of SI, planning, and related symptoms in cycling patients. Current standards of care for suicidality do not include assessment of cyclical symptom changes, and no diagnostic codes are available to describe PME of underlying disorders; clinicians should consider the use of daily ratings to test clinical hypotheses about the role of the menstrual cycle in triggering or exacerbating suicide risk and related symptoms in their ovulating patients. These findings may inform the development of targeted biological treatments to address the impact of the menstrual cycle on acute suicide risk. Ultimately, understanding and addressing sex-specific factors in suicide may reduce the disparities in female risk for suicidal thoughts and behaviors.

## Supplementary information

Supplemental Material
