# Supplementary material for: Effects of acute estradiol and progesterone on perimenstrual exacerbation of suicidal ideation and related symptoms: a crossover randomized controlled trial
Source: Transl Psychiatry. 2022 Dec 30;12:528. doi: 10.1038/s41398-022-02294-1 (PMC9803670; doi:10.1038/s41398-022-02294-1)
Supplement: Supplementary file 1 — Supplemental Material [file 41398_2022_2294_MOESM1_ESM.docx]

**SUPPLEMENTAL MATERIAL**

**Supplemental Figure 1**. Log Estradiol and Progesterone Values Across Days Since Positive LH Test, by Condition (EP vs. PBO).


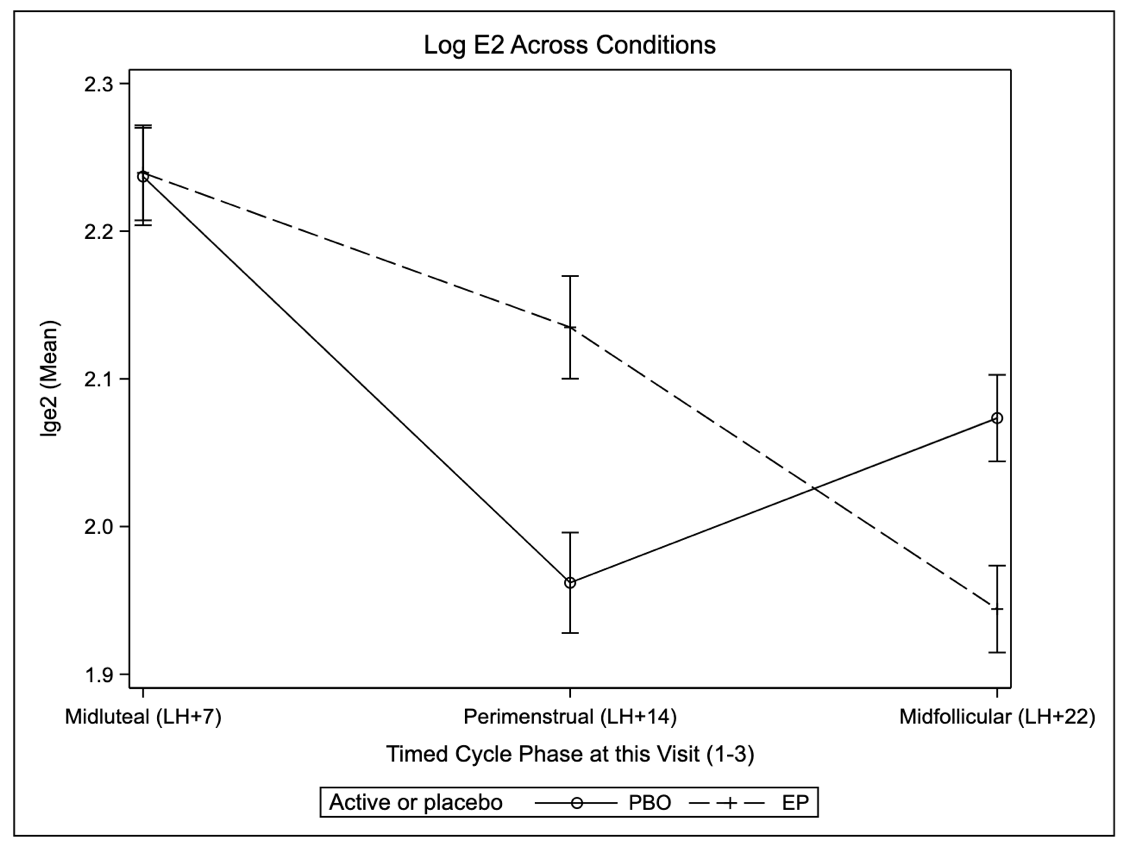


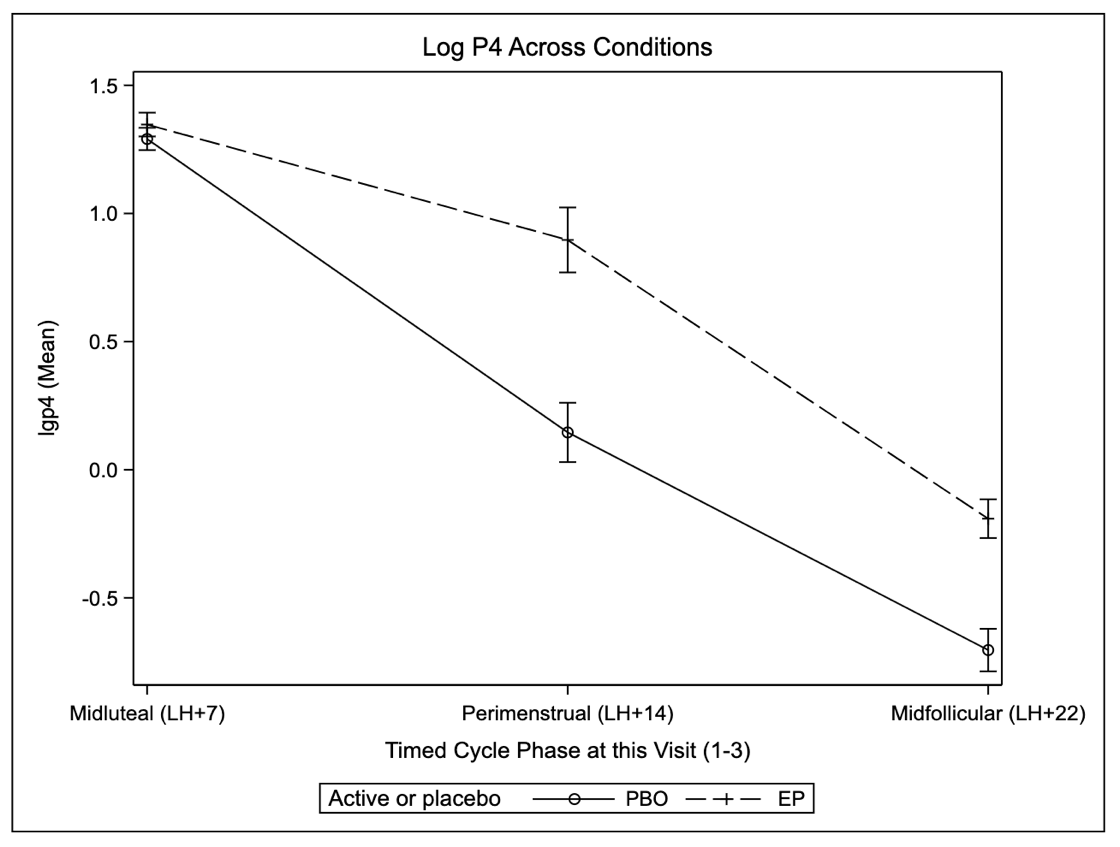


**
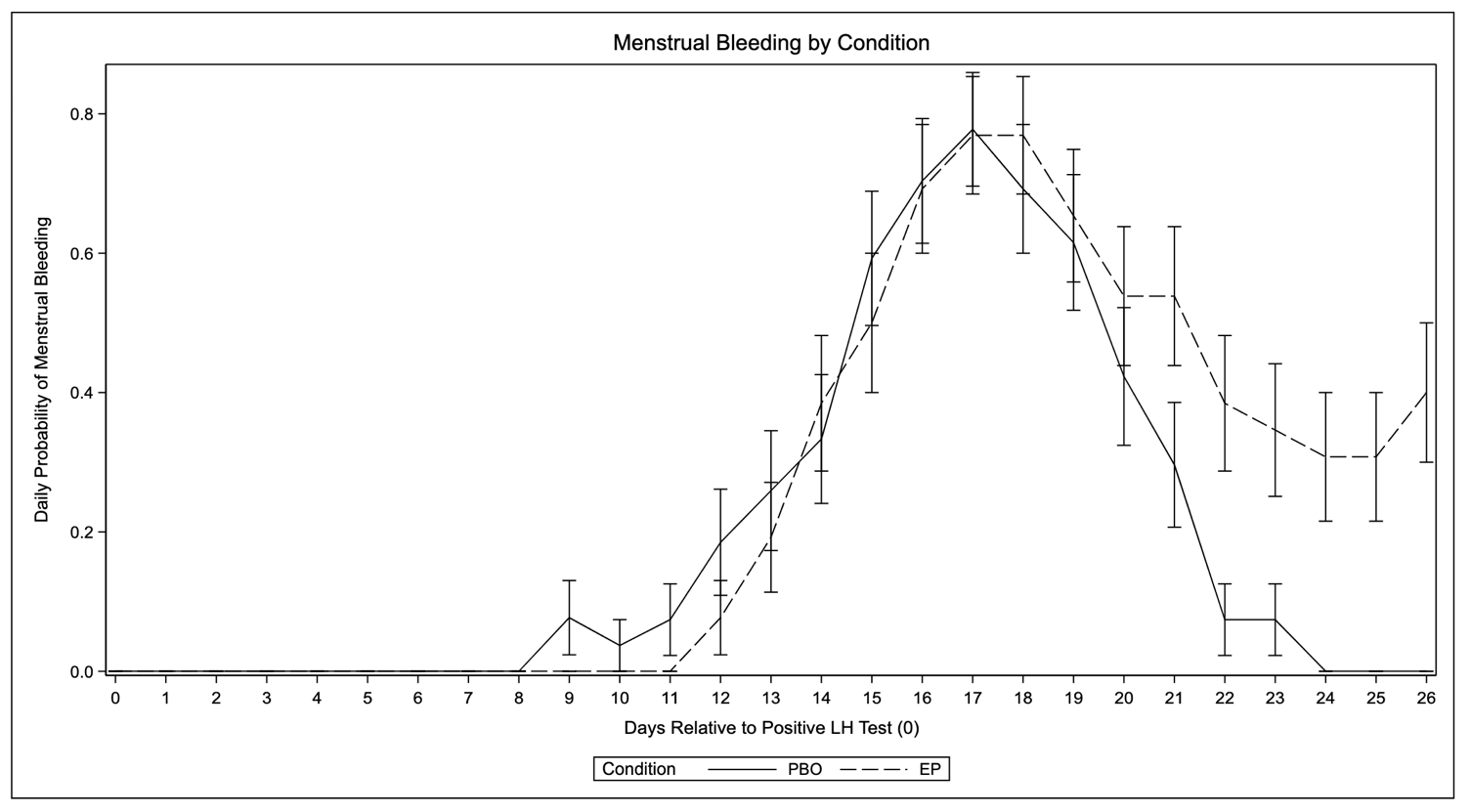
Supplemental Figure 2.** Probability of Menstrual Bleeding Values Across Days Since Positive LH Test, by Condition (EP vs. PBO).

**Supplemental Figures 3**. Graphical Depictions of Secondary and Exploratory Outcomes by Condition and Phase. *Note: Asterisks indicate significant Condition × Phase interaction effects.*


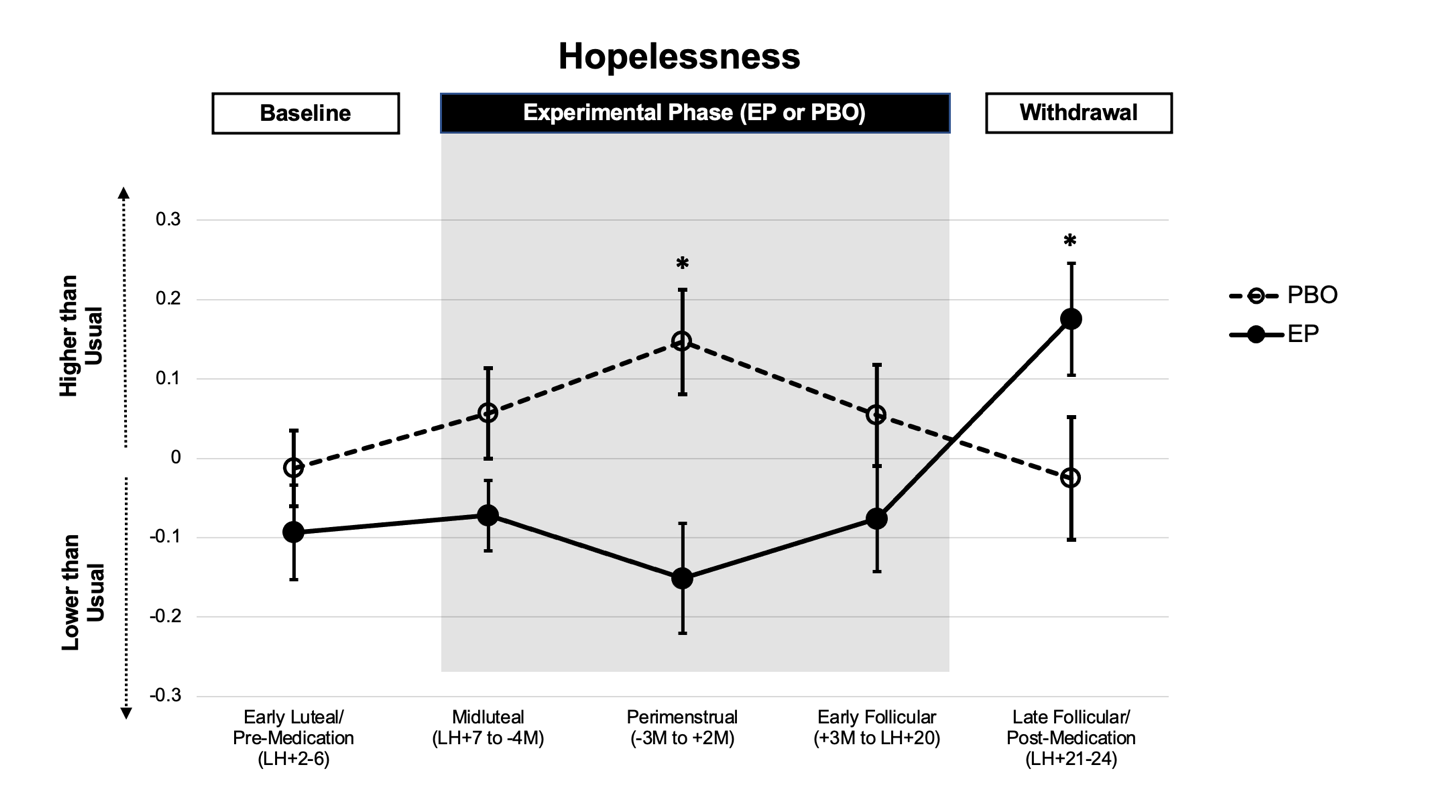

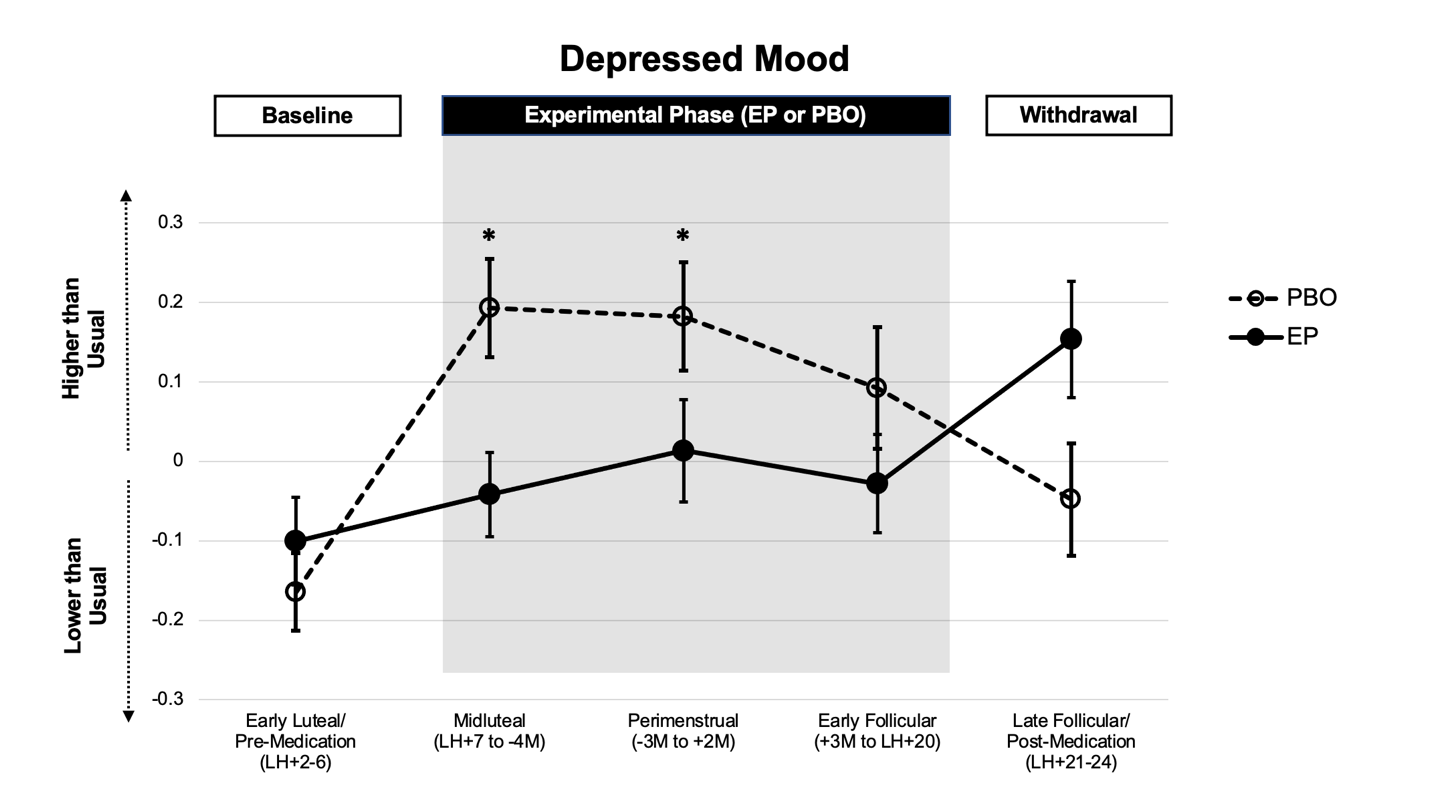


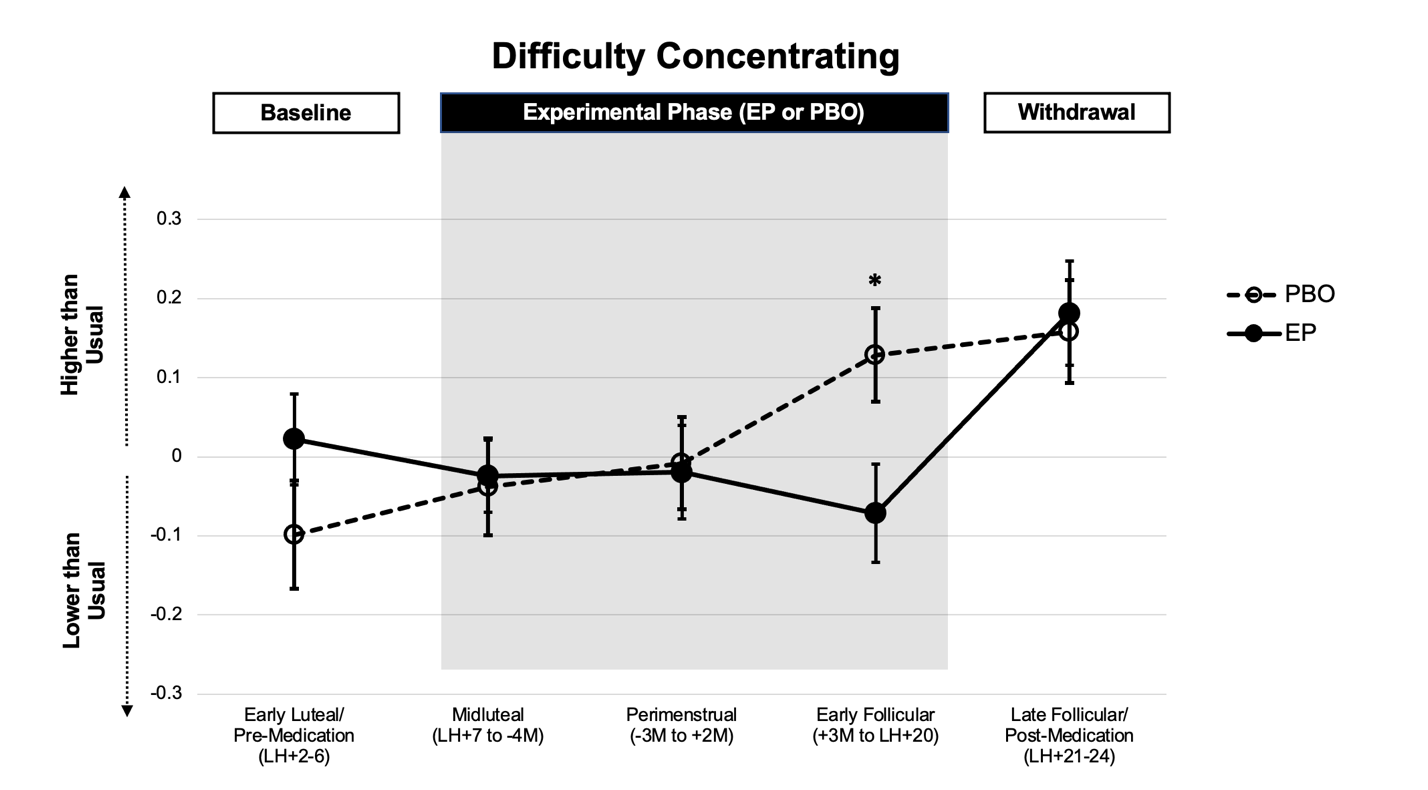

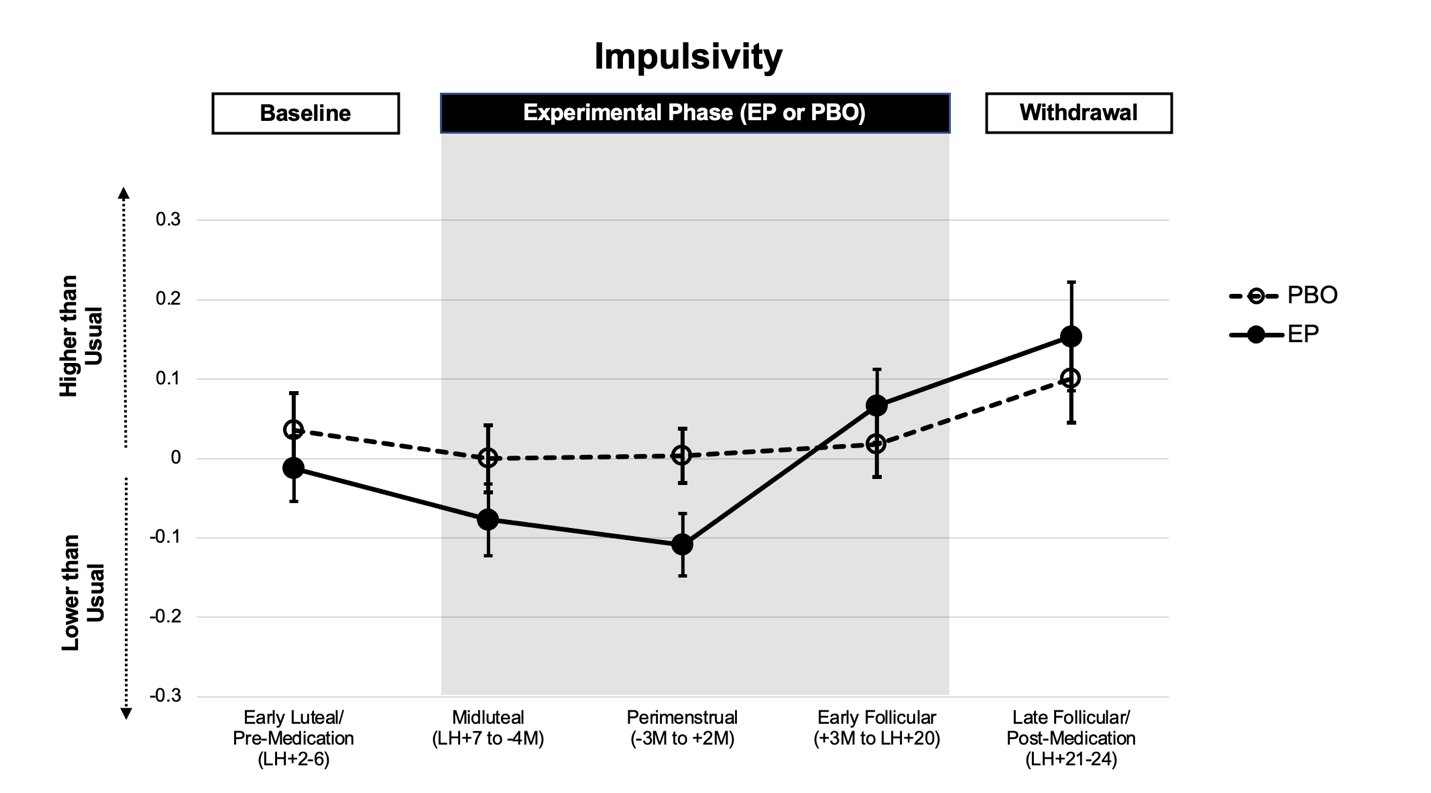


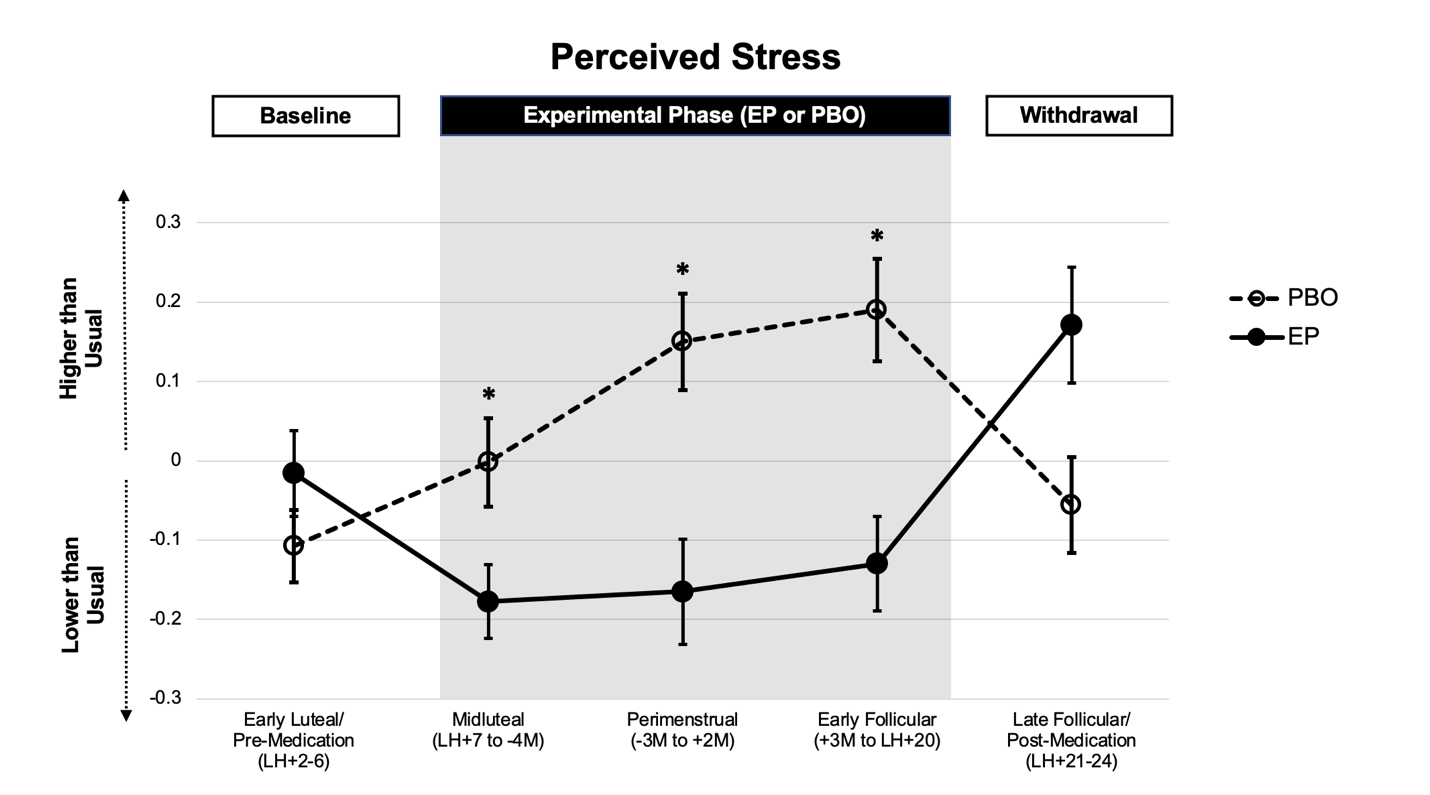

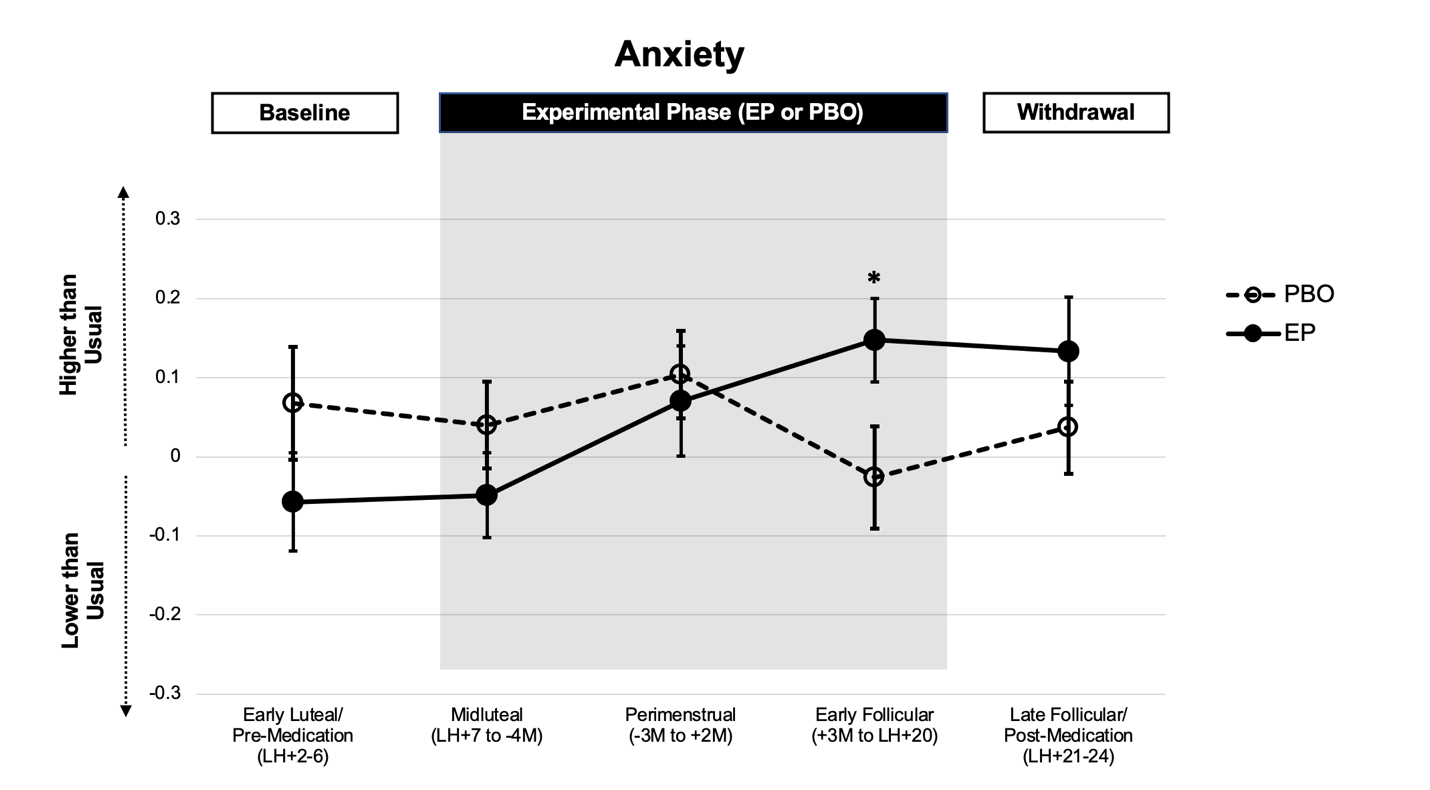


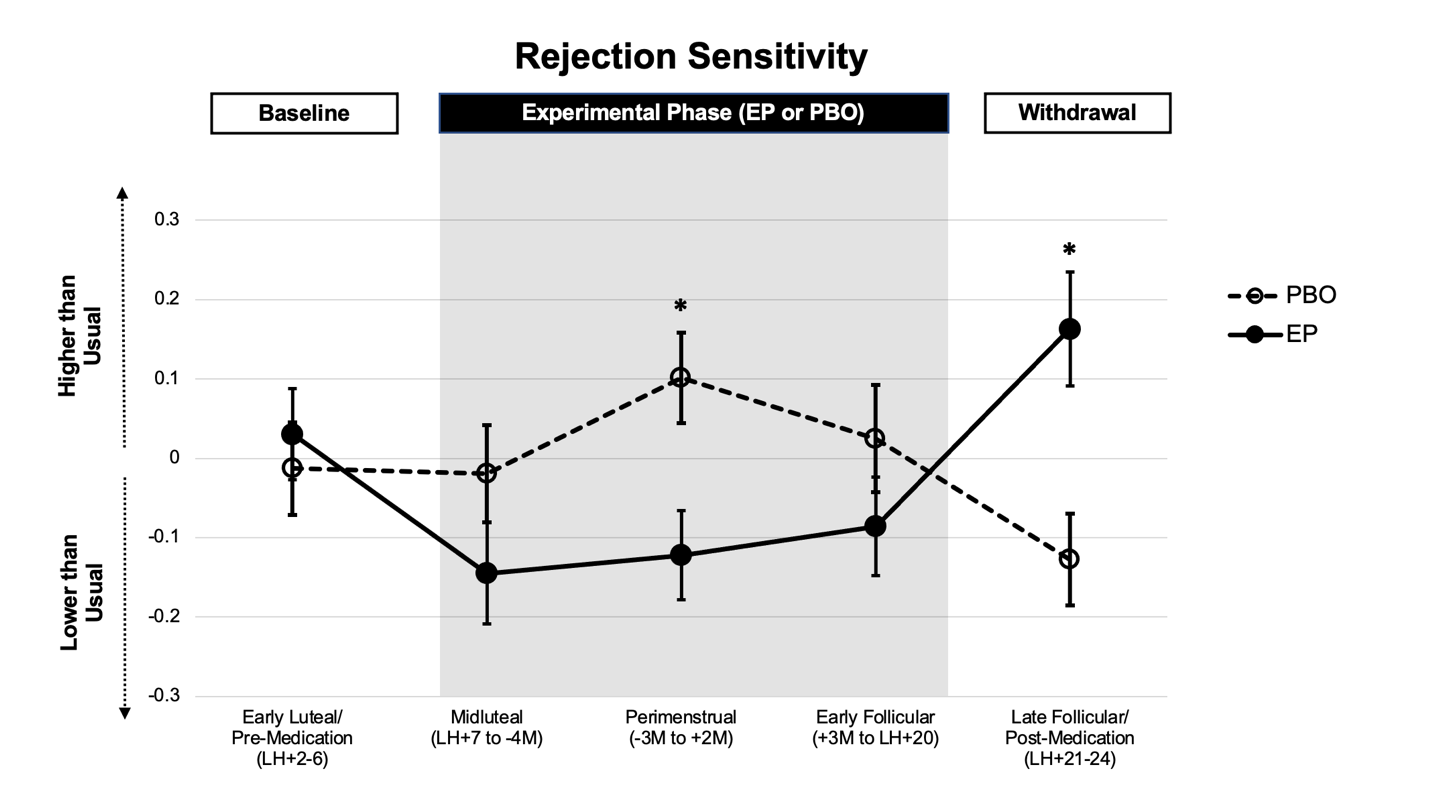
**
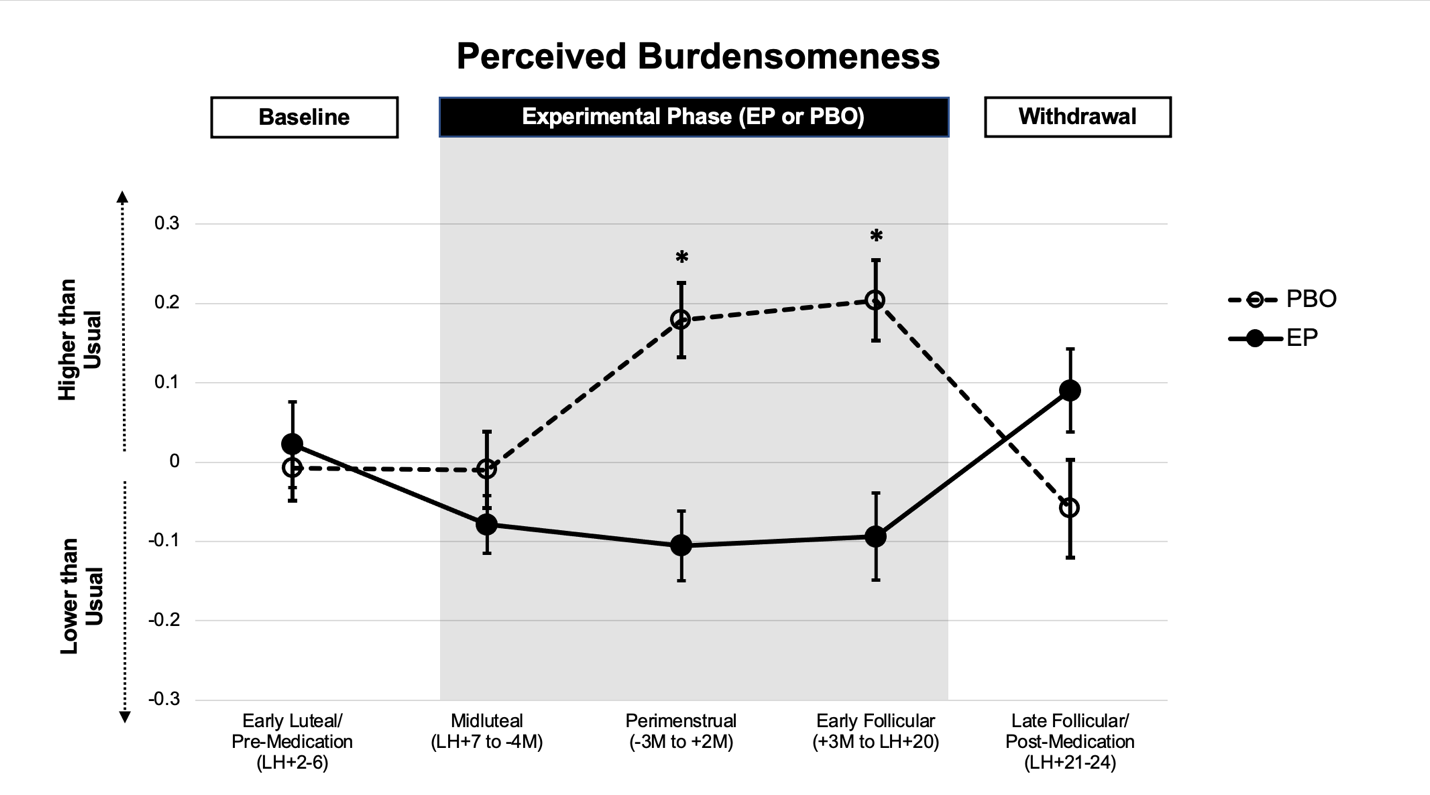
**

**
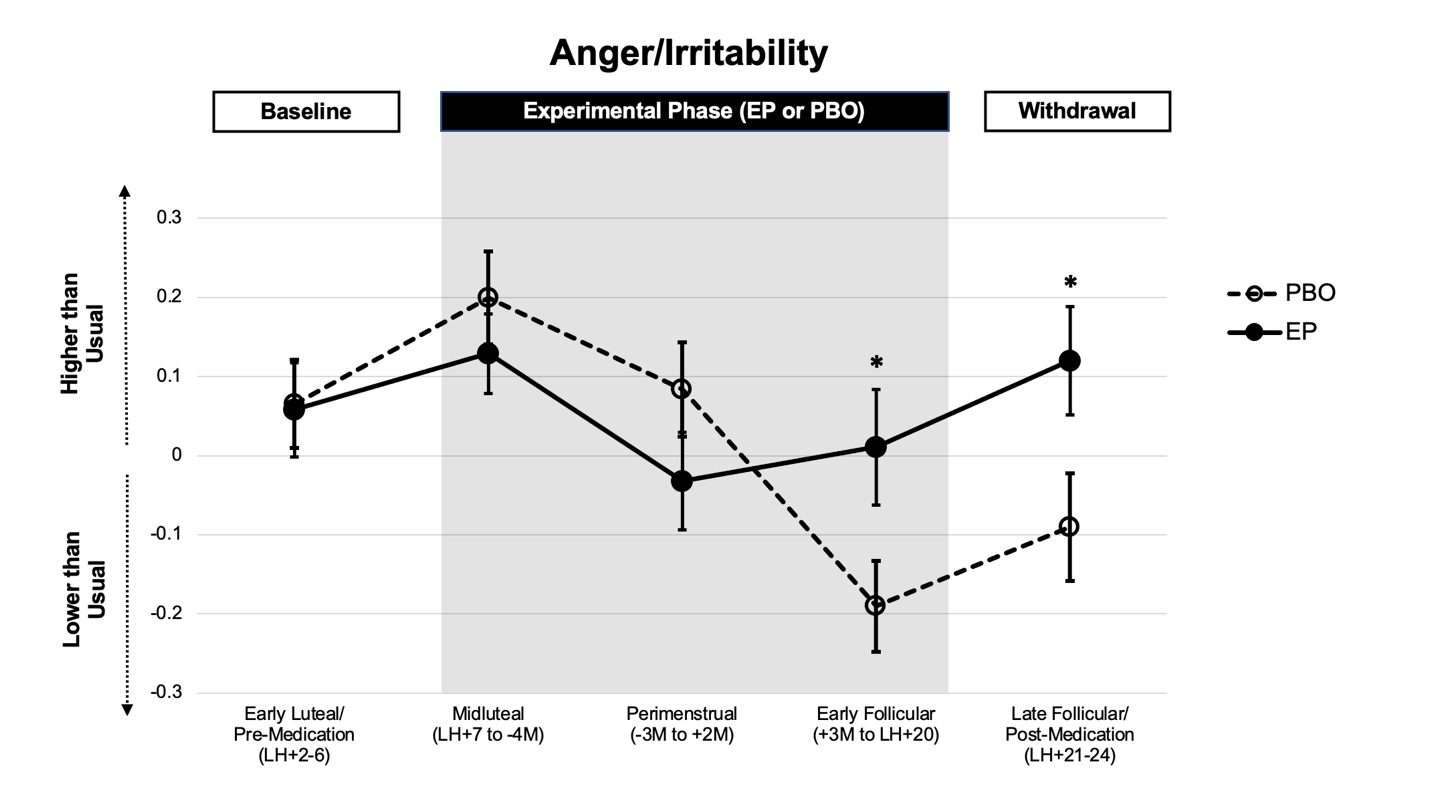
**
